# Intravenous Oxycodone versus Intravenous Morphine in Cancer Pain: A Randomized, Open-Label, Parallel-Group, Active-Control Study

**DOI:** 10.1155/2017/9741729

**Published:** 2017-10-31

**Authors:** Kyung-Hee Lee, Jung-Hun Kang, Ho-Suk Oh, Moon-Ki Choi, Byoung-Yong Shim, Young-Jun Eum, Hye-Jeong Park, Jin-Hyong Kang

**Affiliations:** ^1^Department of Hematology/Oncology, Yeungnam University Hospital, Daegu, Republic of Korea; ^2^Department of Internal Medicine, Gyeongsang National University Hospital, Jinju, Republic of Korea; ^3^Department of Hematology/Oncology, Gangneung Asan Hospital, Gangneung, Republic of Korea; ^4^Center for Colorectal Cancer, National Cancer Center, Ilsan, Republic of Korea; ^5^Department of Medical Oncology, St. Vincent's Hospital, Suwon, Republic of Korea; ^6^Department of Medical Affairs, Mundipharma Korea Ltd, Seoul, Republic of Korea; ^7^Department of Medical Oncology, Seoul St. Mary's Hospital, Seoul, Republic of Korea

## Abstract

**Objective:**

To compare efficacy and safety of intravenous continuous infusion of oxycodone with morphine in patients with cancer pain.

**Methods:**

A 5-day, randomized, open-label, exploratory study at 6 sites in the Republic of Korea. Sixty-six adults aged ≥19 years with moderate-to-severe cancer pain (Numeric Rating Scale [NRS] ≥ 4) were enrolled. The study group received intravenous (IV) oxycodone, and the comparator group received IV morphine which were titrated depending on pain intensity. The efficacy endpoint is change in average NRS score from baseline to Day 5. Other assessments included worst, current, and average pain intensity; patient satisfaction; medication dose; and adverse events.

**Results:**

Both groups achieved >50% reduction in average pain intensity: from “moderate” at baseline (oxycodone versus morphine: 6.0 ± 1.8 versus 5.9 ± 1.4) to “mild” at Day 5 (2.5 ± 1.8 versus 2.8 ± 1.6). While this reduction was similar between groups (3.5 ± 2.2 versus 3.1 ± 1.8, *P* value = 0.562), oxycodone achieved faster pain relief (average pain: 3.0 ± 1.6 versus 3.9 ± 1.6, *P* value = 0.020) on Day 2 and significant NRS reductions for worst pain on Day 2 (*P* value = 0.045) and current pain on Day 2 (*P* value = 0.035) and Day 5 (*P* value = 0.020) compared to morphine. Patient satisfaction, adverse events, and adverse drug reactions were similar for both groups.

**Conclusions:**

For Asian patients with cancer pain, IV oxycodone is faster acting and showed similar analgesic efficacy and safety profiles as IV morphine. This trial is registered with Clinicaltrials.gov NCT02660229.

## 1. Introduction

Patients with cancer can suffer from excruciating pain especially in cases with metastatic, advanced, or terminal disease. The prevalence of pain in patients with cancer exceeds 50% for all cancer types [1, 2]. Cancer pain is recognized as an important health issue as it can suppress the activities of the respiratory, digestive, and urinary systems and induce hormonal changes which can affect physiological functions leading to the failure of normal functions [3, 4]. In Asia, this pain, often of moderate-to-severe intensity, is often undertreated, and patients continue to suffer [2]. The pain can be alleviated with analgesics through intravenous (IV), intramuscular, or oral administration; however, proper pain control is often hard to achieve [5]. Strong analgesics such as oxycodone and morphine are used to treat moderate-to-severe pain where weak opioids failed to provide relief, guided by the 3-step analgesic ladder approach for the prescription of pain relief drugs [6].

Among the administration methods for analgesics, IV infusion is widely used for the management of cancer pain in Korea. IV administration has particular advantages in the form of faster onset of pain relief and more predictable pharmacokinetics compared to other routes of drug administration [7]. Strong opioid analgesics, such as morphine, fentanyl, and alfentanil, which are commonly used for moderate-to-severe pain, are given through IV administration. However, these opioid analgesics act on the central nervous system causing sedation, discomfort, and pruritus and other side effects such as respiratory depression, nausea, and vomiting [8, 9].

Morphine has been the treatment of choice for moderate-to-severe cancer pain. However, its use is often hindered by its unpredictable onset of action, interindividual variability in dose requirements and response [10], and physician concerns about addiction [11]. Oxycodone has been recommended as an alternative to morphine for the treatment of cancer pain [10, 12, 13]. Oxycodone is a semisynthetic opioid, which is synthesized from thebaine. First developed in 1917, its pharmacological action is considered to be similar to morphine and has been used clinically since [14, 15]. There are three opioid receptors in the central nervous system, that is, μ, δ, and κ receptors. Oxycodone, like morphine, acts on the central nervous system via the μ receptor and additionally on the κ receptor (κ-2b receptor) [16–18]. A number of studies, which compared oxycodone with morphine or fentanyl, reported oxycodone as the more effective analgesic: in a study of 40 patients who underwent surgery and received either morphine or oxycodone intravenously, oxycodone exhibited a more rapid effect in alleviating pain at a lower dose compared to morphine [19], and in a study of 78 patients who were given oxycodone or fentanyl immediately after surgery, oxycodone showed a longer duration of action and better analgesic effect than fentanyl [20]. In such postsurgical pain management, oxycodone and morphine are generally given at a dose ratio of 2 : 3 [19]. However, in a double-blind study where oxycodone and morphine were administered intravenously at the same dose immediately after a surgery, the two drugs were found to be equivalent in terms of their analgesic effects [21], and when given for a further 24 hours, the dose and analgesic effect between oxycodone and morphine were also similar.

Studies on pain management using IV administration methods in patients with cancer are somewhat lacking unlike the postsurgical pain management, which have been actively researched. The earliest double-blinded, crossover study comparing oxycodone and morphine as analgesics for cancer pain reported that their analgesic effects were equivalent when administered orally, and when administered intravenously, the IV dose of oxycodone was 30% higher than that of morphine [22]. More nausea was observed in the patients who were given morphine, and further, symptoms of hallucination were also observed only in such patients. Such morphine-induced delirium was alleviated by switching the treatment from morphine to oxycodone [23]. However, this study was conducted on non-Asians. It is already known that the frequency of alleles of the CYP2D6 enzyme which metabolizes such drugs in the liver differs by ethnicity [24]. While Caucasians typically have a higher percentage of functional CYP2D6 alleles compared to Asians, the available evidence is still inconclusive on whether CYP2D6 metabolism does impact analgesic effectiveness [7, 25]. Hence, an exploratory study is necessary to confirm whether oxycodone would present the same efficacy and safety profiles in patients of Asian ethnicity who suffer from cancer pain. The objective of this study is to compare the efficacy and safety of oxycodone and morphine when administered by IV continuous infusion in Korean patients with cancer pain.

## 2. Materials and Methods

This study was designed as a multicenter, randomized, open-label, active-controlled exploratory study to evaluate the change in pain intensity measured by the Numerical Rating Scale (NRS, 0–10 points: 0 = no pain and 10 = worst pain) from baseline to Day 5 (120 hours) when administering oxycodone or morphine through IV continuous infusion in patients with cancer pain (clinicaltrials.gov: NCT02660229). It was conducted in accordance with Korean Good Clinical Practice (KGCP) [26] and International Conference on Harmonisation (ICH) [27] guidelines, complied with the rights and safety of patients under the Declaration of Helsinki and approved by the Institutional Review Boards (IRB) of all institutions. All patients provided written informed consent prior to their participation in the study.

### 2.1. Study Design

**Figure 1:**
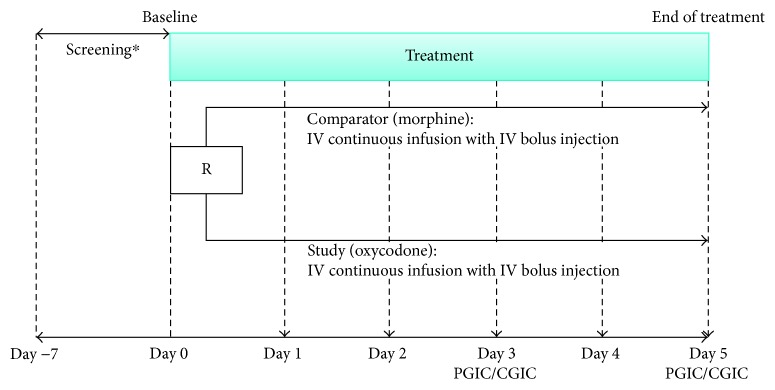
Study design and visit schedule. ^∗^At screening, patients can be randomized if average pain intensity NRS score during previous 7 days is ≥4. IV = intravenous, NRS = Numerical Rating Scale (0–10 points, 0 = no pain and 10 = worst pain), PGIC/CGIC = Patient Global Impression of Change/Clinical Global Impression of Change, R = randomization.

The study had two arms: the study (oxycodone) group received oxycodone (OxyNorm®, EP 10 mg/1 ml or 20 mg/2 ml ampoules) and the comparator (morphine) group received morphine (BC Morphine Sulfate hydrate injection®, 5 mg/5 ml or 30 mg/2 ml ampoules) and adhered to the intention-to-treat (ITT) principles (Figure 1). Data on baseline information, cancer status, medical history, and prior medication history were collected, and patients who met the inclusion/exclusion criteria were randomized to either the oxycodone or morphine group in a 1 : 1 ratio within each site. The investigator administered the pertinent drug (oxycodone or morphine) following the order in the randomization list when patients were enrolled and was not blinded to the treatment allocation. A unique identification number for each subject was assigned. When a subject was withdrawn during the study, his/her identification number was withdrawn as well.

At Day 0 (baseline), the use of existing opioid analgesics was discontinued, and the pain was stabilized with IV bolus injection of oxycodone or morphine at a dose determined by the investigator based on the dose of the previous analgesics. Where the patient was using a strong opioid for the first time, 2 mg of oxycodone or morphine was initially administered by IV bolus injection to stabilize the pain.

The oxycodone/morphine medication was administered by IV continuous infusion for 5 days (120 hours). All medication doses were diluted in 0.9% normal saline. The dose administered was adjusted at the investigator's discretion according to the subject's pain intensity, and the relevant time, date, and dose were recorded in the chart. Subjects in both groups received prophylactic laxatives, and the study drugs were combined with an antiemetic to alleviate nausea and vomiting symptoms.

### 2.2. Patients

The study enrolled patients with cancer aged ≥19 years who experienced moderate-to-severe pain (NRS ≥ 4) over the past 7 days as verbally confirmed at screening, were hospitalized, or were scheduled for hospitalization and not planned to be discharged during the study period. Key exclusion criteria included patients who have reached the opioid analgesic dose (oral morphine dose 195 mg/day, oral oxycodone dose 130 mg/day, or patch fentanyl dose 75 μg/hour) for cancer pain prior to screening, or had a medical history of hypersensitivity to oxycodone or morphine or other opioid analgesics, or clinically significant respiratory disorder or severe respiratory dysfunction. Also excluded were patients on monoamine oxidase inhibitors; with moderate-to-severe hepatic impairment, that is, ALT or AST > 3.0 upper limit of normal (ULN), total bilirubin > 1.5 × ULN, and respiratory depression or hypotension; receiving anticancer therapy that may affect pain control measurement, at the discretion of the investigator, or scheduled for radiotherapy during the study period; or with clinically significant cardiovascular or renal dysfunction or pregnancy.

### 2.3. Study Assessments

#### 2.3.1. Pain Intensity

To adjust the dose of the study drugs for appropriate pain management, investigators checked pain intensity at screening and baseline and continuously after medication administration. At screening, patients were asked to indicate the average pain intensity (using NRS) experienced for the past 7 days. At baseline (Day 0), Day 1, Day 2, Day 3, Day 4, and Day 5, the patient was asked to indicate current, worst, and average pain experienced over the past 24 hours using the NRS, and these were recorded. Verbal measurements were allowed without using visual data.

The primary efficacy endpoint is the change in average NRS pain score from baseline (Day 0) to Day 5 (120 hours). Secondary endpoints include change in the worst, current, and average NRS scores from baseline to Day 1, Day 2, Day 3, and Day 4 and change in the worst and current NRS scores from baseline to Day 5.

#### 2.3.2. Total Administered Drug Dose

The dose of the study drugs (IV infusion + bolus injection) intravenously administered was checked and recorded every day. The dose administered from baseline to each assessment time point was based on records on the chart, and in the event of dose change/end of treatment, the pertinent date and time and dose were recorded. Total administered dose of the study drugs (mg) = IV infusion (mg/hr) ∗ [(end date − start date) ∗ 24 + (end time − start time)] + bolus injection (mg).

#### 2.3.3. Treatment Satisfaction

On Day 3 and Day 5 (end of the study), overall analgesic treatment satisfaction regarding pain was assessed by investigators using a 7-point Clinical Global Impression of Change (CGIC) scale (1 = very much improved to 7 = very much worse) [28, 29] and by patients using a 7-point Patient Global Impression of Change (PGIC) scale (1 = very much improved to 7 = very much worse) [28].

#### 2.3.4. Collection of Safety Data

Physical examination was performed, and all vital signs were measured each day. Clinical laboratory tests and ECG were conducted at baseline and at Day 5. Patients were asked to report all adverse events (AEs), and these were confirmed through interview and history taking. A list of possible AEs was provided to the investigators to assist them in this process. The severity of the AE was assessed using a 3-point grade system, that is, (1) mild (causes mild discomfort but does not interfere with daily activities), (2) moderate (causes significant discomfort and interferes with daily activities), and (3) severe (prevents normal daily activities). The information on AEs included onset and resolution dates, severity and outcome, action taken and causal relationship to the study drugs, and name of suspected drug other than the study drugs. Safety was assessed based on AEs, vital signs, physical examination, clinical laboratory tests, and ECGs. All safety data were collected and documented in the case report form (CRF).

#### 2.3.5. Statistical Analysis

As this was an exploratory study, we targeted to enrol a total of 66 patients (33 in each group). This number sufficiently allowed for an estimated 10% dropout rate and normality assumptions on the data and is also used in a number of similar studies [30, 31]. Descriptive statistics, that is, frequency, mean, and SD, were used for demographic, cancer, and health data. For the continuous data, the differences between the study and comparator groups were tested using the two-sample *t*-test or Wilcoxon rank sum test (where normality cannot be assumed), and for categorical data, for example, sex and cancer history, the analysis was conducted using the chi-square test or Fisher's exact test.

The mean, SD, and percent change at each time point from baseline were computed for the study and comparator groups for the NRS score, change in NRS scores, and cumulative administered dose. A two-sample *t*-test or Wilcoxon rank sum test was conducted to test for differences between groups. A paired *t*-test or Wilcoxon signed rank test was used to analyze within-group differences in the change of NRS scores at each time point. Additional analyses on the cumulative proportion of subjects in each group who achieved at least 30% and 50% reduction in pain levels on each day of study were computed. A chi-square test was used to analyze the between-group difference in proportions.

The frequency and proportion for the CGI-C and PGI-C scores on Day 3 and Day 5 were summarized for each group, and Fisher's exact test was conducted to test the difference between groups. All analyses were performed using the SAS™ statistical analysis software (SAS Institute, Cary, NC, USA, version 9.4), and statistical significance was evaluated at 0.05 levels.

## 3. Results

**Figure 2:**
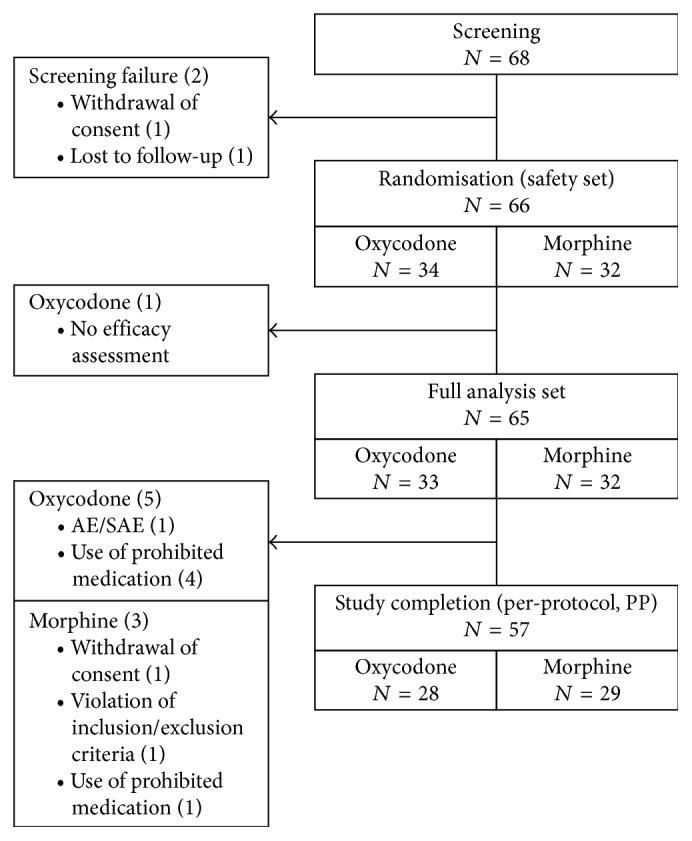
Flow of patients through the trial.

The study was conducted from 3 September 2015 to 24 July 2016 at 6 sites. A total of 68 patients were screened. Of these, 2 failed the screening, and the remaining 66 patients, that is, safety set (SS) consisting of 34 patients for the oxycodone group and 32 in the morphine group, were randomized for treatment (Figure 2). One patient from the oxycodone group without an efficacy assessment was excluded. The full analysis (FA) set consisted of 65 patients (oxycodone: 33; morphine 32). Eight patients were further excluded. The reasons for exclusion are shown in Figure 2. A total of 57 patients completed the study (PP set, oxycodone: 28; morphine: 29).

Patients' demographic and cancer characteristics are shown in Table 1. About 6 in 10 patients were males. The average age was 66.6 ± 9.1 years for the oxycodone group and 64.1 ± 13.0 years for the morphine group. There were no differences in their age and cancer duration profiles. The cancer-type profiles of the two groups were different (*P* value = 0.042); pancreatic and gastric cancers were most common in the oxycodone group while gastric, lung, and colorectal cancers were more represented in the morphine group. Most (83.1%) patients had concurrent illnesses. Almost half (49.2%) of the patients had chemotherapy from 14 days before screening till end of study, but none had radiation therapy. Nearly all patients had prior medication (other than anticancer therapy), and all of them had concomitant medication.

### 3.1. Pain Intensity Scores

**Figure 3:**
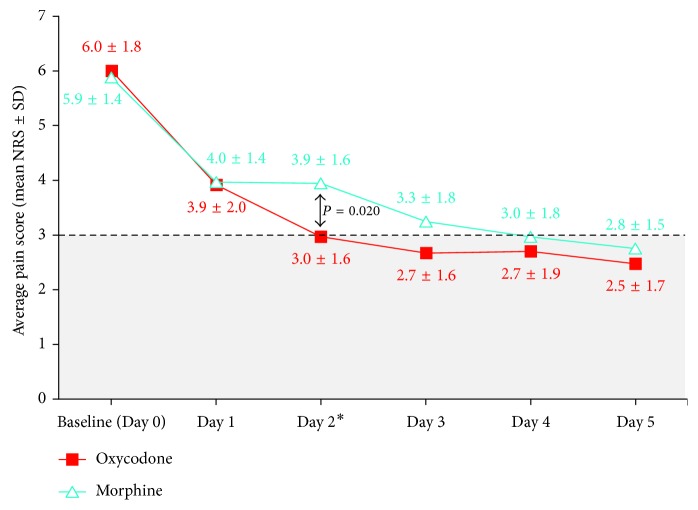
Average NRS pain scores. ^∗^Difference between average pain scores is significant. NRS = Numerical Rating Scale (0–10 points, 0 = no pain and 10 = worst pain).

Figure 3 shows the average pain experienced by patients for each treatment for each day of the study. At baseline (Day 0), the average NRS pain scores were similar for both groups in the FA set (oxycodone versus morphine: 6.0 ± 1.8 versus 5.9 ± 1.4, *P* value = 0.963) and, similarly, at the end of the study (Day 5, 2.5 ± 1.8 versus 2.8 ± 1.6, *P* value = 0.565). However, on Day 2, there is a significant difference in the average pain scores (3.0 ± 1.6 versus 3.9 ± 1.6, *P* value = 0.020). Patients who were given IV oxycodone reported average NRS scores of 3 or lower from Day 2 onwards compared to Day 4 for the morphine group. Similar observations were also recorded for the current pain scores.

Table 2 shows the changes in worst, current, and average pain scores in each group, compared to baseline on a day-by-day basis. These changes were significant (*P* value < 0.001) from Day 1 onwards with the oxycodone group having the largest one-day decrease in worst, current, and average pain scores of 1.8, 2.0, and 2.1 points, respectively, from baseline to Day 1. On Day 5, both groups achieved >50% reductions in average NRS pain scores, that is, from “moderate” to “mild” pain as compared to baseline. There was no difference in this reduction between the two groups (*P* value = 0.553). The PP set also showed similar results. The worst pain scores, at baseline, were 8.0 ± 1.8 and 7.6 ± 1.7 (*P* value = 0.353) for the oxycodone and morphine groups, respectively. These decreased to 4.5 ± 2.1 and 5.1 ± 2.3 (*P* value = 0.263) on Day 5. Between the two groups, oxycodone achieved significantly better pain relief for the worst pain recorded on Day 2 (−2.9 ± 2.7 (oxycodone) versus −1.7 ± 2.2 (morphine); *P* value = 0.045) and current pain on Day 2 (−2.7 ± 2.7 (oxycodone) versus −1.2 ± 2.0 (morphine); *P* value = 0.035) and Day 5 (−3.4 ± 2.6 (oxycodone) versus −1.9 ± 1.6 (morphine); *P* value = 0.020) (Table 2).

**Figure 4:**
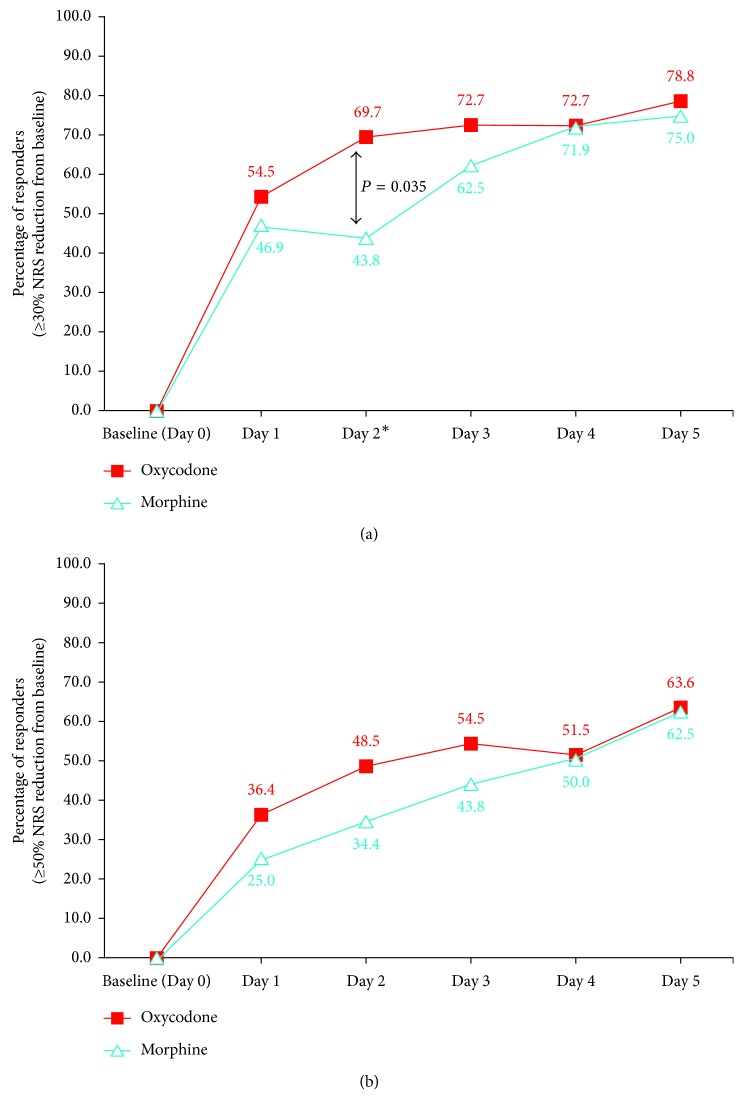
Percentage of responders based on (A) ≥30% and (B) ≥50% NRS reduction from baseline. ^∗^Difference between percentages of responders is significant. NRS = Numerical Rating Scale.

Figure 4 shows the percentage of patients who achieved at least 30% and 50% reduction in average pain compared to baseline on each day of treatment for each group. There was a significant difference in the percentages of responders with *at least* 30% reduction in pain for the oxycodone group compared to the morphine group on Day 2 (Figure 4, 69.7% versus 43.8%, *P* value = 0.035).

### 3.2. Treatment Satisfaction

There were no differences in the treatment satisfaction scores reported by patients (PGIC) and investigators (CGIC) for both drugs on Day 3 and Day 5. By Day 3, most patients (≥95.3%) reported some improvement in pain relief regardless of the pain medication. The investigators also reported similar observations. Similar results were also observed in the PP set.

### 3.3. Treatment Dose

The mean cumulative doses of oxycodone and morphine groups of the FA set at the end of the study (Day 5) were 226.8 ± 110.4 mg and 226.6 ± 135.1 mg (*P* value = 0.996), respectively. There were no differences in the cumulative medication doses given to each group on a daily basis during the study period.

### 3.4. Adverse Events

Table 3 shows the incidence of AEs in the SS set. They were similar in both groups: 85.3% (29/34 patients, 64 events) in the oxycodone group and 81.3% (26/32 patients, 58 events) in the morphine group (*P* value = 0.660). This was also the case for the incidence of serious AEs: 8.8% (3/32 patients, 3 events) and 6.3% (2/32 patients, 2 events) in the oxycodone and morphine groups, respectively.

The most commonly reported AE in both groups was gastrointestinal disorders (oxycodone: 22/34 = 64.7%; morphine: 16/32 = 50.0%, Table 3) due mostly to constipation (oxycodone: 13/34 = 38.2%; morphine: 6/32 = 18.8%) and nausea (oxycodone: 10/34 = 29.4%; morphine: 8/32 = 25.0%). For constipation, the incidence of AEs that were drug-related was similar between the two groups, that is, oxycodone, 53.8% (7/13), and morphine, 50.0% (3/6). Besides, 55.6% of patients had reported gastrointestinal disorders at the start of the study. Most (87.7%) of the AEs are of the lowest severity, that is, mild. There was a difference in the unexpected AEs with more (29) events affecting 16 patients (50.0%) in the morphine group compared to 12 events in the oxycodone group affecting 9 patients (26.5%) (*P* value = 0.049). The nature of these disorders (by system-order-class) is shown in Table 3. Two patients in the oxycodone group dropped out due to AEs (nausea and hyperhidrosis) while there were none from the morphine group. There were no differences in the incidence of adverse drug reaction events between the groups (41.2% versus 34.4%, *P* value = 0.569).

## 4. Discussion

Our exploratory study showed that oxycodone and morphine both achieved at least 50% reduction in average intensity, from “moderate pain” at baseline to “mild pain” at Day 5, for our Asian patients suffering moderate-to-severe cancer pain. These results are in agreement with those of a 2016 study that compared strong opioids, including oxycodone and morphine, for pain control in Italian patients with cancer. Oxycodone and morphine reduced pain intensities by a similar order of magnitude, that is, average NRS scores decreased from 6.0 ± 1.5 and 6.0 ± 1.3 to 2.9 ± 1.9 and 2.8 ± 2.0, respectively, on Day 7 [13]. In our study, both investigators and patients in both the oxycodone and morphine groups agreed that the pain condition has improved when asked on Day 3 and Day 5 compared to baseline. However, we noted that patients who were given IV oxycodone achieved an average pain score of NRS ≤ 3 by Day 2. This is earlier when compared to Day 4 for patients on IV morphine. The NRS = 3 pain score target was found to be the median personalized pain goal across patients suffering different degrees of pain in studies on cancer pain and provides a tangible objective of the patient's expectations of his/her cancer pain [32, 33] and served as a useful benchmark to assess the efficacy of the treatment. Compared to our baseline pain scores, it represents a 50% reduction and exceeds the minimal (33%) clinically important difference in pain perception.

Patients who were given oxycodone reported greater reductions in the pain scores from baseline for worst pain on Day 2 and current pain on Day 2 and Day 5 as compared to morphine. They also recorded larger single-day reductions on Day 1 on all the three pain intensity measures. However, these daily observations are limited and would need to be corrected for multiple comparisons. The effectiveness of a centrally acting analgesic drug depends on its bioavailability in the central nervous system, particularly, the brain. Its rate of action is dependent on the transport of that drug across the blood-brain barrier (BBB). The influx rate across the BBB is much more rapid for oxycodone compared to morphine, and the extent of this difference has been estimated to be sixfold faster [34, 35]. This could explain the faster onset of analgesic action of oxycodone on Day 2 compared to morphine. Both opioids have different effects in the sensitized pain system: oxycodone achieved greater pain relief from heat and electrical stimulation in the skin, muscle, and esophagus of healthy patients [36]. Although the μ-opioid-binding affinity of oxycodone is lesser compared to morphine, additional antinociceptive effect was achieved via its κ-opioid receptors. This also has a faster onset of analgesic effect, as observed in our patients [18, 37].

The cumulative doses of drug administered intravenously were similar in both our study groups, demonstrating that a 1 : 1 potency for oxycodone to morphine can be achieved in Asian patients with cancer pain. This potency ratio has previously been estimated for oral administration to range from 1 : 1 to 1 : 2.2, with the consensus that oral oxycodone is about 1.5–2 times as potent as oral morphine [16]. Oral opioids are typically used in cancer pain management. However, subcutaneous administration has a more rapid onset of analgesia compared to oral administration, achieving peak plasma concentrations within 15–30 minutes and has been recommended as an alternative route where oral administration is not possible [38].

Pain is a personal and subjective experience, and a number of measures have been developed to describe the level of pain, for example, pain intensity difference, pain relief, % of maximum total pain relief (TOTPAR), and sum of pain intensity difference (SPID) [39]. The timing of pain assessments during the study could influence the size of effects or differences that a study detects. In our pilot study, pain measurements were recorded every day using 3 measures of pain (current, average, and worst pain), providing a comprehensive profile of pain reduction over the study period. We noted that reporting differences between the two treatments using the change in mean pain values over time as a measure of efficacy of the treatment drug can be limited by the variability in baseline values or by large changes in a few patients. Farrar et al. [39, 40], in a review of various measures of pain, proposed reporting the proportion of patients who have clinically important improvement in their pain care as an alternative. We examined the cumulative pain reduction by measuring the proportion of patients who achieved at least 30% and 50% pain reduction during the study period. In our study, there was a significant difference in the responder rate at ≥30% level of reduction in pain on Day 2, with 69.7% of patients on oxycodone compared to 43.8% for morphine (Figure 4), demonstrating the faster onset of pain relief of oxycodone. When using absolute pain intensity, the recommended cutoff was 2 for pain difference or 33%, where the percentage change was measured. Oxycodone also achieved the proposed 2-point reduction in absolute terms earlier, that is, on Day 1 compared to Day 3 for morphine.

The incidence of AEs is >80% for both groups. However, there were no significant differences between oxycodone and morphine groups in the incidence of AEs, or associated adverse drug reactions. A meta-analysis of studies on the use of strong opioids for cancer pain management reported similarly high incidences regardless of the opioid used [41]. The analysis also revealed that nausea and constipation, AEs which were most reported by our patients, are typically reported AEs in patients with cancer-related pain. However, the dose effect, where higher rates of AEs were observed with higher doses of opioid given, was mostly observed in morphine cohorts [41]. In our study, patients who were given morphine had a significantly higher number of unexpected AEs compared to oxycodone. Corli et al. [13] reported that while the adverse effects of morphine were similar to other strong analgesics including oxycodone, neurotoxicity in morphine was greater and was often ameliorated by rotating patients out of morphine into oxycodone.

We are aware that this small sample size and the short duration of our exploratory study did not allow a formal evaluation of efficacy of the study drugs due to potential statistical bias and the limited time for the full effects of the drugs to be realized. An open-label study design was chosen as the study aimed to evaluate the two drugs under conditions approximating normal clinical use. This may cause some bias in the subjective assessments especially from the investigators as an active comparator was used. We recognized this to be a limitation of a nonblinded design. However, the route of administration for both drugs was similar, that is, IV, and together with the randomization, may minimize some of the bias.

While there are some negative effects on the use of strong opioids such as tolerance and abuse [42], achieving adequate control of cancer pain is important as many patients discontinue treatment as a result of insufficient pain relief or adverse events [43]. Wang et al. [31], in a meta-study on the efficacy and tolerability of oxycodone in an Asian population, found pain intensity scores of oxycodone to be superior compared to other strong opioids for the management of moderate-to-severe cancer-related pain in China with no differences in the incidence of side effects such as dizziness, vomiting, sleepiness, pruritus, anorexia, and dysuria. Morphine has been the gold standard for treatment of severe pain. Baek et al. [44] showed that high-dose oxycodone could be safely and effectively used for pain management in Korean patients with cancer and can have a role in the management of moderate-to-severe pain especially where drug tolerance or side effects become an issue.

## 5. Conclusions

Our study showed that oxycodone and morphine, administered by IV infusion for patients with moderate-to-severe cancer pain in an Asian population, have similar efficacy and safety profiles. Oxycodone was found to be faster acting and can be a good alternative to morphine.

## Figures and Tables

**Table 1:** Patient characteristics and medical conditions.

Patient characteristic/medical condition (full analysis set)	Oxycodone (*N* = 33)	Morphine (*N* = 32)	*P* value
Sex, male, *n* (%)	21 (63.6)	22 (68.8)	0.663^∗^
Age, mean ± SD, years	66.6 ± 9.1	64.1 ± 13.0	0.379^†^
Age distribution, *n* (%)			
19–39 years, *n* (%)	0 (0)	2 (6.3)	0.516^§^
40–49 years, *n* (%)	1 (3.0)	2 (6.3)	
50–59 years, *n* (%)	5 (15.2)	6 (18.7)	
≥60 years, *n* (%)	27 (81.8)	22 (68.7)	
Weight, mean ± SD, kg	58.9 ± 10.1	59.2 ± 12.2	0.925^†^
Cancer duration,^∗∗^ median (range), months	7.3 (0.1–72.0)	14.5 (0.1–149.0)	0.401^‡^
<1 year, *n* (%)	20 (62.5)	14 (43.7)	0.392^§^
≥1 to <5 years, *n* (%)	10 (31.2)	14 (43.7)	
≥5 years, *n* (%)	2 (6.3)	4 (12.6)	—
Unknown	1^∗∗^	—	—
Cancer type			
Pancreatic cancer	8 (24.2)	3 (9.4)	**0.042** ^§^
Gastric cancer	7 (21.2)	6 (18.8)	—
Gall bladder/biliary tract cancer	3 (9.1)	0 (0)	—
Lung cancer	2 (6.1)	5 (15.6)	—
Liver cancer	2 (6.1)	0 (0)	—
Breast cancer	2 (6.1)	0 (0)	—
Colorectal cancer	1 (3.0)	5 (15.6)	—
Other cancers	8 (24.2)	13 (40.6)	—
Cancer stage^††^			
I	0 (0)	0 (0)	0.432^§^
II	2 (6.1)	0 (0)	—
III	4 (12.1)	2 (6.5)	—
IV	27 (81.8)	29 (93.5)	—
Unknown	—	1^††^	—
Concurrent illnesses, *n* (%)	28 (84.5)	26 (81.3)	0.699^∗^
Had chemotherapy 14 days prior to screening till end of study, *n* (%)	14 (42.4)	18 (56.3)	0.265^∗^
Prior medication excluding anticancer therapy	32 (97.0)	31 (96.9)	>0.999^§^

^∗^ *χ* ^2^-test; ^†^2-sample *t*-test; ^§^Fisher's exact test; ^‡^Wilcoxon rank sum test; ^∗∗^Duration of cancer history (months) = (initiation date of study treatment − date of cancer diagnosis)/30; oxycodone group: 1 subject with unknown cancer duration was excluded; ^††^morphine group: 1 subject with unknown cancer stage was excluded.

**Table 2:** Change in average, worst, and current NRS^∗^ pain scores from baseline (Day 0).

Change in NRS from Day 0 (full analysis set)	Oxycodone (*N* = 33)		Morphine (*N* = 32)		*P* value
	*n*	Mean ± SD	*n*	Mean ± SD	
Change in average pain score^∗^ from Day 0					
On Day 1	33	−2.1 ± 2.5	32	−1.9 ± 1.7	>0.999^†^
On Day 2	33	−3.0 ± 2.4	32	−1.9 ± 2.0	0.065^†^
On Day 3	33	−3.3 ± 2.3	32	−2.6 ± 2.1	0.212^†^
On Day 4	33	−3.3 ± 2.2	32	−2.9 ± 2.0	0.612^†^
On Day 5	33	−3.5 ± 2.2	32	−3.1 ± 1.8	0.562^†^
Percentage change					
On Day 5, %	33	−56.7 ± 27.3	32	−51.9 ± 25.2	0.553^†^
Change in worst pain score^∗^ from Day 0					
On Day 1	26	−1.8 ± 2.6	25	−1.8 ± 2.7	0.723^†^
On Day 2	26	−2.9 ± 2.7	25	−1.7 ± 2.2	**0.045** ^†^
On Day 3	26	−3.2 ± 2.6	25	−2.8 ± 2.2	0.703^†^
On Day 4	26	−3.2 ± 2.7	25	−2.7 ± 2.4	0.633^†^
On Day 5	26	−3.5 ± 2.5	25	−2.5 ± 2.3	0.152^§^
Change in current pain score^∗^ from Day 0					
On Day 1	26	−2.0 ± 3.0	25	−1.2 ± 1.8	0.541^†^
On Day 2	26	−2.7 ± 2.7	25	−1.2 ± 2.0	**0.035** ^†^
On Day 3	26	−2.9 ± 2.7	25	−1.5 ± 2.1	0.072^†^
On Day 4	26	−2.8 ± 2.6	25	−1.8 ± 1.8	0.293^†^
On Day 5	26	−3.4 ± 2.6	25	−1.9 ± 1.6	**0.020** ^§^

^∗^NRS (LOCF): 0–10, 0—least pain, 10—most pain; ^†^Wilcoxon rank sum test; ^§^2-sample *t*-test; LOCF = last observed carried forward, NRS = Numerical Rating Score.

**Table 3:** Incidence of adverse events.

Safety set	Oxycodone (*N* = 34)			Morphine (*N* = 32)			*P* value (*χ*2 test)
	Incidence, *n* (%)	95% CI^§^	No. of events	Incidence, *n* (%)	95% CI^§^	No. of events	
		(lower limit, upper limit)			(lower limit, upper limit)		
Adverse events	29 (85.3)	(68.9, 95.1)	64	26 (81.3)	(63.6, 92.8)	58	0.660
(i) Gastrointestinal disorders	22 (64.7)	(17.4, 50.5)	30	16 (50.0)	(9.3, 40.0)	23	—
(ii) Nervous system disorders	7 (20.6)	(6.7, 34.5)	7	5 (15.6)	(3.5, 29.0)	5	—
(iii) General disorders and administration site	6 (17.7)	(0.0, 10.3)	7	6 (18.8)	(0.0, 16.2)	6	—
(iv) Skin and subcutaneous tissue disorders	5 (14.7)	(0.1, 15.3)	5	4 (12.5)	(0.8, 20.8)	4	—
(v) Other disorders	15 (44.1)	—	15	16 (50.0)	—	20	—
Unexpected adverse events	9 (26.5)	(12.9, 44.4)	12	16 (50.0)	(31.9, 68.1)	29	**0.049**
(i) Blood and lymphatic system disorders	3 (8.8)	(1.9, 23.7)	3	1 (3.1)	(0.1, 16.2)	1	**—**
(ii) Gastrointestinal disorders	2 (5.9)	(0.7, 16.7)	2	5 (15.6)	(5.3, 32.8)	5	**—**
(iii) Metabolism and nutrition disorders	2 (5.9)	(0.7, 19.7)	2	2 (6.3)	(0.8, 20.8)	3	**—**
(iv) Injury, poisoning, and procedural complications	2 (5.9)	(0.7, 19.7)	2	0	(0.0, 10.9)	0	**—**
(v) Respiratory, thoracic, and mediastinal disorders	1 (2.9)	(0.1, 15.3)	1	4 (12.5)	(3.5, 29.0)	5	**—**
(vi) Infections and infestations	1 (2.9)	(0.1, 15.3)	1	2 (6.3)	(0.8, 20.8)	2	**—**
(vii) Investigations	1 (2.9)	(0.1, 15.3)	1	1 (3.1)	(0.1, 16.2)	3	**—**
(viii) General disorders and administration site	0	(0.0, 10.3)	0	5 (15.6)	(5.3, 32.8)	5	**—**
(ix) Renal and urinary disorders	0	(0.0, 10.3)	0	3 (9.4)	(2.0, 25.0)	3	**—**
(x) Other disorders	0	—	0	2 (6.3)	—	2	**—**
Dropouts^∗^	2 (5.9)	(0.7, 19.7)	7	0 (0.0)	(0.0, 10.9)	0	0.493^†^
Serious adverse events	3 (8.8)	(1.9, 23.7)	3	2 (6.3)	(0.8, 20.8)	2	>0.999^†^
Adverse drug reaction^‡^	14 (41.2)	(24.7, 59.3)	20	11 (34.4)	(18.6, 53.2)	17	0.569
Serious adverse drug reaction	0 (0.0)	(0.0, 10.3)	0	0 (0.0)	(0.0, 10.9)	0	—
Unexpected adverse drug reaction	0 (0.0)	(0.0, 10.3)	0	1 (3.1)	(0.1, 16.2)	1	0.485^†^

^∗^Dropouts caused by adverse events are subjects whose reason for dropout was “difficult to perform the study due to AE or SAE”; ^†^exact test; ^§^the 95% CI was calculated using the exact method; ^‡^adverse drug reactions are adverse events collected as “certain,” “probable/likely,” “possible,” “conditional/unclassified,” or “unassessable/unclassifiable” for the causal relationship to the study drug.
